# Intraindividual Variability in Domain-Specific Cognition and Risk of Mild Cognitive Impairment and Dementia

**DOI:** 10.1155/2013/495793

**Published:** 2013-12-22

**Authors:** Leslie Vaughan, Iris Leng, Dale Dagenbach, Susan M. Resnick, Stephen R. Rapp, Janine M. Jennings, Robert L. Brunner, Sean L. Simpson, Daniel P. Beavers, Laura H. Coker, Sarah A. Gaussoin, Kaycee M. Sink, Mark A. Espeland

**Affiliations:** ^1^Department of Social Sciences and Health Policy, Wake Forest University School of Medicine, Medical Center Boulevard, Winston-Salem, NC, USA; ^2^Wake Forest University, Winston-Salem, NC, USA; ^3^National Institute on Aging, Baltimore, MD, USA; ^4^Family and Community Medicine, University of Nevada School of Medicine, Reno, NV, USA

## Abstract

Intraindividual variability among cognitive domains may predict dementia independently of interindividual differences in cognition. A multidomain cognitive battery was administered to 2305 older adult women (mean age 74 years) enrolled in an ancillary study of the Women's Health Initiative. Women were evaluated annually for probable dementia and mild cognitive impairment (MCI) for an average of 5.3 years using a standardized protocol. Proportional hazards regression showed that lower baseline domain-specific cognitive scores significantly predicted MCI (*N* = 74), probable dementia (*N* = 45), and MCI or probable dementia combined (*N* = 101) and that verbal and figural memory predicted each outcome independently of all other cognitive domains. The baseline intraindividual standard deviation across test scores (IAV Cognitive Domains) significantly predicted probable dementia and this effect was attenuated by interindividual differences in verbal episodic memory. Slope increases in IAV Cognitive Domains across measurement occasions (IAV Time) explained additional risk for MCI and MCI or probable dementia, beyond that accounted for by interindividual differences in multiple cognitive measures, but risk for probable dementia was attenuated by mean decreases in verbal episodic memory slope. These findings demonstrate that within-person variability across cognitive domains both at baseline and longitudinally independently accounts for risk of cognitive impairment and dementia in support of the predictive utility of within-person variability.

## 1. Introduction

There is continued interest in identifying optimal and novel cognitive predictors of dementia of Alzheimer's type (AD) as both diagnostic and predictive entities [1–5]. Although it may be difficult to discriminate AD in its early preclinical stages from normal cognitive aging, it is believed that preclinical markers of AD may be present in older adults for many years before the appearance of clinical symptoms [6, 7]. While neuropsychological tests of both memory and executive function (EF) have been used to discriminate normal cognitive aging from both mild cognitive impairment (MCI) and AD [8, 9], the role of intraindividual variability among cognitive domains is not well understood.

The preclinical onset of dementia is marked by decreases in both overall global and domain-specific cognition [2, 10–13], in particular episodic memory and executive function (EF). Decrements in verbal episodic memory are generally identified as one of the earliest markers of cognitive decline [14–16], followed by global changes in multiple cognitive domains that occur several years before the onset of dementia  [6, 17, 18]. The role of EF is not well characterized as a predictor of dementia, although it has been explored in relation to time of AD onset. Grober et al. [7] examined change points in linear mixed models of cognitive variables over time in persons who later developed AD and found that declines in performance on tests of EF accelerated 2-3 years before diagnosis, whereas declines in performance on tests of verbal episodic memory (sum of immediate recall across 3 learning trials) accelerated 7 years before diagnosis.

Most studies of cognitive predictors of dementia focus on interindividual differences in mean performance, yet increases in within-person intraindividual variability (IAV) also may signal the preclinical onset of dementia. Intraindividual variability refers to changes within persons, and measures of IAV may include across neuropsychological tests of cognitive domains administered on a single occasion (IAV Cognitive Domains), across trials within computerized tasks (IAV Tasks), and longitudinal change in IAV among neuropsychological tests of cognitive domains (IAV Time). The focus of this study is on IAV Cognitive Domains and IAV Time, as described below. For example, Holtzer et al. [19] examined within-person across neuropsychological test variability (IAV Cognitive Domains) of individually administered neuropsychological tests (Free and Cued Selective Reminding Test, Digit Symbol, Vocabulary) and incident dementia and found that a nested Cox proportional hazards model of IAV Cognitive Domains marginally improved the prediction of incident dementia compared to interindividual differences in mean performance on neuropsychological tests and significantly increased the sensitivity for predicting dementia within one year, but the Holtzer et al. study did not address IAV Time. Although IAV Time over the lifespan may be high in normal aging individuals (e.g., [20]), there is little research on how longitudinal changes in within-person variability across neuropsychological tests might predict dementia (although see [21]). Overall, studies of various types of IAV are consistent with the possibility that increased IAV in cognition, independent of interindividual differences in mean performance, may reflect a breakdown in attentional control and memory systems [22–24]. Therefore, IAV Cognitive Domains and IAV Time may improve the preclinical or early detection of incident dementia.

We examined data from the Women's Health Initiative Study of Cognitive Aging (WHISCA) [16, 25, 26] to determine if IAV Cognitive Domains and IAV Time predict risk of MCI and incident dementia, addressing two main questions. First, we examined whether IAV Cognitive Domains predicts risk of MCI and dementia above and beyond mean overall and domain-specific interindividual differences in cognition. Using a similar strategy, we next examined if IAV Time predicts risk of MCI and dementia. We hypothesized that IAV Cognitive Domains should predict risk of MCI and incident dementia above and beyond interindividual differences in cognition, in-line with other studies that have found increased IAV among cognitive domains in persons with dementia [19, 22], with the expectation that verbal episodic memory might partially account for this result. Additionally, we predicted that IAV Time should predict risk of MCI and dementia above and beyond baseline interindividual differences in cognition [21] and baseline intraindividual differences in cognition.

## 2. Methods

The Women's Health Initiative Memory Study (WHIMS) [27] began enrolling its 7,479 participants from the parent Women's Health Initiative hormone therapy (HT) trials [28] in May, 1996. These women were between 65 and 79 years of age at initial screening, were appropriate candidates for postmenopausal HT, and were free of dementia as assessed with a standard protocol. Women without a uterus were randomly assigned with equal probability to take one daily tablet that contained either 0.625 mg of conjugated equine estrogens (CEE: Premarin, Wyeth-Ayerst Philadelphia, PA) or a matching oral placebo in the WHI CEE-Alone trial. In a similar manner, women with a uterus were randomly assigned to take one daily tablet that contained either 0.625 mg of CEE with 2.5 mg medroxyprogesterone acetate (MPA: PremPro, Wyeth-Ayerst Philadelphia, PA) or placebo in the WHI CEE+MPA trial.

The Women's Health Initiative Study of Cognitive Aging (WHISCA) enrolled 2,304 of these participants from 14 of the WHIMS clinical sites beginning in September, 1999 [16]. These women had been randomly assigned to WHI treatments for a mean (standard deviation) of 3.0 (0.7) years prior to enrollment in WHISCA and were free of probable dementia and mild cognitive impairment (MCI) at baseline according to standardized assessments, as described below. Participants were administered a cognitive battery and 3MSE [29] screening annually for a mean (range) of 5.3 (0.7 to 8.4) years through September, 2007. The National Institutes of Health and Institutional Review Boards for all participating institutions approved protocols and consent forms. Informed written consent was obtained from all participants.

The WHI CEE+MPA trial ended in July, 2002 [30] and the CEE-Alone trial ended in February, 2004 [31]; however, WHIMS annual follow-up, with reconsenting, continued until September, 2007. Results from all cognitive assessments that occurred to the end of this extended follow-up are presented in this paper.

### 2.1. Cognitive Assessments

A battery of cognitive measures was administered annually to WHISCA participants by trained administrators [16]. It included the primary mental abilities vocabulary test of verbal knowledge (PMA Vocabulary) [32], the Benton Visual Retention Test of short-term figural memory (BVRT) [33], a modified version of the California Verbal Learning Test of verbal memory (CVLT) [16, 34, 35], the digit span forward and backward tests of attention and working memory [36], the card rotations test of spatial ability [37], and the letter and semantic fluency tests of verbal fluency [38, 39]. Fluency tasks, widely used to test executive function, are thought to depend on strategic retrieval, initiation of action, inhibition of previously dominant responses, and ability to switch search strategies [40, 41]. Additionally, a finger tapping test was administered to assess fine motor speed [42].

The cognitive battery was administered in face-to-face interviews by certified test administrators. Quality control was maintained through recertification of test administrators twice during the first year and annually thereafter.

### 2.2. Classification of Probable Dementia

Detailed descriptions of the WHIMS protocol for detecting MCI and probable dementia has been published [13, 27, 43]. All WHIMS participants were administered the (3MSE) [29] annually. If women scored below preset standard cut-points based on education level (≤88 for ≥9 years of education; ≤80 for ≤8 years) on the 3MSE, they were given a complete neuropsychiatric evaluation that included the Consortium for the Establishment of a Registry for Alzheimer's Disease (CERAD) [44, 45] battery of neuropsychological tests and standardized questions about acquired cognitive and functional impairments to participants and a knowledgeable friend or family member. Using a standardized protocol developed by the WHIMS Clinical Coordinating Center, a boardcertified local dementia specialist (i.e., neurologist, geropsychiatrist, or geriatrician) reviewed all available data and administered a clinical evaluation. The local dementia specialists then classified each woman into 1 of 3 groups: probable dementia based on clinical diagnostic criteria for Alzheimer's disease from the Diagnostic and Statistical Manual of Mental Disorders, Fourth Edition (DSM-IV) [17], WHIMS MCI, or no impairment. Criteria for WHIMS MCI were based on accepted criteria at the time of WHIMS initiation. This was defined operationally as poor performance (10th or lower percentile on the CERAD norms on at least 1 CERAD test), a report from the designated informant of some functional impairment, no evidence of a psychiatric disorder or medical condition that could account for the decline in cognitive function, and an absence of adjudicated dementia. Those suspected of having probable dementia underwent a noncontrast CT brain scan and laboratory blood tests to rule out possible reversible causes of cognitive decline, and local dementia specialists were required to provide the most probable etiology. All data on the WHIMS participants except the local dementia expert's classification were then submitted to a centralized adjudication committee consisting of dementia experts at the WHIMS coordinating center at Wake Forest University School of Medicine for final classification of no impairment, MCI, or probable dementia. The committee reviewed all the probable dementia cases and a random sample of nonprobable dementia cases without knowledge of the local dementia expert's diagnosis and achieved high reliability with the latter. Our analyses focus on the WHISCA cohort, a subset of the WHIMS participants, who were all cognitively normal at baseline and include all cases of adjudicated dementia that were triggered by clinic-based 3MSE testing during follow-up.

### 2.3. Statistical Analyses

All analyses were performed using SAS 9.3 (SAS Institute Inc., Cary, NC, USA). The significance level for all tests was set at 0.05. The results of multiple analyses are presented. Because the primary predictors were IAV Cognitive Domains and IAV Time, we considered other predictors exploratory, and no further multiple comparison corrections were made. Test scores from each of the cognitive domains were standardized by converting them to *z*-scores, using the means and standard deviations (SDs) at WHISCA enrollment. An average of the *z*-scores from the seven tests was used as an overall measure of global cognitive function. We also created two composite scores to test the independent contributions of EF and verbal episodic memory: EF was calculated as the average of the *z*-scores of verbal fluency (letter and semantic fluency tests of verbal fluency [38, 39]) and working memory (the digit span forward and backward tests of attention and working memory [36]); verbal episodic memory was calculated as the average of the *z*-scores of learning/immediate recall, short-delay free recall, and long-delay free recall subscales from the CVLT [34]. Cronbach's alpha was calculated for the EF and verbal episodic memory composites. The measure of intraindividual variability (IAV Cognitive Domains) was the standard deviation (SD) among the seven cognitive tests at baseline. The longitudinal measure of intraindividual variability (IAV Time) was calculated as follows. First, a regression function of each cognitive test against time was calculated for each woman. Then, the SD of the seven slopes of the cognitive tests was calculated as IAV Time for that woman. In a similar fashion, slopes of overall cognitive function, EF, and verbal episodic memory were calculated.

Cox proportional hazards models were used to evaluate the relative risk of each predictor. The person time at risk was defined as the time since the WHISCA baseline cognitive function evaluation to the time of diagnosis of MCI, probable dementia, and MCI or probable dementia combined. All models were adjusted for age, education, and WHI treatment assignment. The first set of Cox models examined whether interindividual differences in mean performance on overall, composite, and domain-specific test scores predicted MCI, probable dementia, and MCI or probable dementia. The second set of Cox models examined the relationship between IAV Cognitive Domains and MCI, probable dementia, and MCI or probable dementia. The third set of Cox models examined the relationship between IAV Time and MCI, probable dementia, and MCI or probable dementia combined, by additionally adjusting for confounding cognitive factors as shown in Table 3. In the full model, we tested whether IAV Time still predicts MCI, probable dementia, and MCI or probable dementia, after adjusting for baseline overall cognition, EF, verbal episodic memory, IAV Cognitive Domains, on overall slope, EF slope, and verbal episodic memory slope. We also compared those women who had only baseline cognitive data to those who had at least 2 cognitive assessments on overall cognition, EF, and verbal episodic memory composites.

## 3. Results

The 2,305 women enrolled in WHISCA were followed with annual 3MSE screening for an average (range) of 5.3 (0.7 to 8.4) years through September, 2007, for an average of 5.6 measurement occasions. At the time of their WHISCA enrollment, women averaged 74.0 (range: 66.9 to 84.5) years of age. The distribution of their education levels was: 5.2% less than high school, 21.4% high school graduates, 41.2% some posthigh school education, and 32.2% college graduates. The cohort included 6.2% African-American women, 1.3% Hispanic/Latina women, 90.1% Caucasian women, and 2.4% women representing other race/ethnicities. The WHI treatment assignments for these women were 30.0% CEE+MPA, 31.7% CEE+MPA placebo, 18.8% CEE alone, and 19.6% CEE alone placebo. A total of *N* = 239 women were excluded from the analyses (*N* = 52 had a WHISCA visit after a cognitive assessment; *N* = 24 had events before their first WHISCA visit; *N* = 13 had missing values on at least one of the seven cognitive measures; *N* = 147 had only a baseline visit and could not be used to calculate slope; and *N* = 3 had missing values on education), with a final sample size of *N* = 2066. Those women who were lost to follow-up (e.g., those who had only WHISCA baseline data; *N* = 147) had lower cognitive scores on each test (all *P* < 0.01), lower overall cognition (*P* < 0.0001), lower EF (*P* = 0.0002), and lower verbal episodic memory composite scores (*P* < 0.0001), with the exception of fine motor speed and the measure of IAV at baseline, IAV Cognitive Domains (all *P* > 0.50). Of the final sample, *N* = 74 women developed MCI, *N* = 45 developed probable dementia, and *N* = 101 had MCI or probable dementia. None of the groups (MCI; probable dementia; MCI or probable dementia) differed from the final sample on HT Arm (all *P* > 0.10) or education (all *P* > 0.10), but all of the groups had greater frequencies of women 75 years and older (MCI 28% versus 12%; probable dementia 38% versus 12%; MCI or probable dementia 33% versus 12%) (all *P* < 0.0001).

Table 1 shows the raw scores from the tests of cognitive function at the first WHISCA visit, for all women (*N* = 2066), women with no cognitive impairment (*N* = 1965), MCI (*N* = 74), probable dementia (*N* = 45), and MCI or probable dementia (*N* = 101). Raw scores were converted to domain-specific *z*-scores for the use in subsequent analyses. Standardized total scores for verbal fluency, CVLT, digits forward and backwards, and fine motor speed for each group are also shown. Additionally, composite measures of overall cognition (the average of the *z*-scores for all cognitive domains), EF (the average of the *z*-scores for verbal fluency and attention and working memory), and episodic verbal memory (the average of the *z-scores* for CVLT learning/immediate recall (CVLT no. correct 3 list A trials), CVLT short-delay free recall (CVLT no. correct list A short-delay free recall), and CVLT long-delay free recall (CVLT no. correct list A long-delay free recall) are reported. Cronbach's alpha for EF and verbal episodic memory composites were 0.66 and 0.72, respectively.

Table 1 compares women classified with no impairment to women with MCI, probable dementia, and MCI or probable dementia over a mean follow-up of 5.3 (2.0) years. Group differences between women with no impairment and MCI were significant on all standardized cognitive measures (all *P* < 0.05) with the exception of letter fluency (*P* = 0.06). IAV Cognitive Domains did not differ significantly between women with no impairment (M = 0.80, SD = 0.26) and women with MCI (M = 0.81, SD = 0.27) (*P* = 0.82). However, IAV Time demonstrated significant differences between women with no cognitive impairment (M = −0.009, SD = 0.51) and women with MCI (M = 0.22, SD = 0.60) (*P* = 0.002). In the women with probable dementia, group differences between women with no impairment and probable dementia were significant on all standardized cognitive measures (all *P* < 0.001), except for digits forward (*P* = 0.08), letter fluency (*P* = 0.21), and finger tapping (all *P* > 0.20). IAV Cognitive Domains differed significantly between women with no impairment (M = 0.80, SD = 0.26) and women with probable dementia (M = 0.92, SD = 0.29) (*P* = 0.004). In addition, IAV Time demonstrated significant differences between women with no cognitive impairment (M = −0.009, SD = 0.51) and women with probable dementia (M = 0.28, SD = 0.59) (*P* = 0.0002). Lastly, group differences between women with no impairment and women with MCI or probable dementia were significant on all standardized cognitive measures (all *P* < 0.05). IAV Cognitive Domains did not differ significantly between women with no impairment (M = 0.80, SD = 0.26) and women with MCI or probable dementia (M = 0.84, SD = 0.27) (*P* = 0.15). However, IAV Time demonstrated significant differences between women with no cognitive impairment (M = −0.009, SD = 0.51) and women with MCI or probable dementia (M = 0.24, SD = 0.60) (*P* = 0.0001).

Interindividual differences in each standardized cognitive domain were significantly related to subsequent risk of MCI (all *P* < 0.05) (Table 2). Controlling for all other cognitive domains, domain-specific scores for verbal memory (HR = 0.32; 95% CI = 0.24, 0.42; *P* < 0.0001) and figural memory (HR = 0.74; 95% CI = 0.58, 0.95; *P* = 0.019) significantly predicted MCI. Overall cognition (the standardized mean of the seven individual tests) was also strongly predictive of dementia (HR = 0.32; 95% CI = 0.25, 0.41; *P* < 0.0001), as were EF (HR = 0.48; 95% CI = 0.37, 0.63; *P* < 0.0001) and verbal episodic memory composites (HR = 0.27; 95% CI = 0.21, 0.35; *P* < 0.0001) (see Table 3). For the probable dementia group, interindividual differences in each standardized cognitive domain were also significantly related to subsequent risk (all *P* < 0.001) with the exception of finger tapping (*P* = 0.50) (Table 2). Controlling for all other cognitive domains, domain-specific scores for verbal memory (HR = 0.26; 95% CI = 0.17, 0.39; *P* < 0.0001) and figural memory (HR = 0.68; 95% CI = 0.50, 0.92; *P* = 0.012) significantly predicted probable dementia, whereas vocabulary (*P* = 0.05) and card rotations were marginal (*P* = 0.06). Overall cognition was also strongly predictive of probable dementia (HR = 0.25; 95% CI = 0.18, 0.35; *P* < 0.0001), as were EF (HR = 0.50; 95% CI = 0.35, 0.70; *P* < 0.0001) and verbal episodic memory composites (HR = 0.22; 95% CI = 0.16, 0.31; *P* < 0.0001) (see Table 3). Lastly, interindividual differences in each standardized cognitive domain were significantly related to the risk of MCI or probable dementia (Table 2) (all *P* < 0.01). Controlling for all other cognitive domains, domain-specific scores for verbal memory (HR = 0.34; 95% CI = 0.27, 0.44; *P* < 0.0001) and figural memory (HR = 0.71; 95% CI = 0.57, 0.87; *P* = 0.001) significantly predicted MCI or probable dementia. Overall cognition was also strongly predictive of MCI or probable dementia (HR = 0.30; 95% CI = 0.24, 0.37; *P* < 0.0001), as were EF (HR = 0.48; 95% CI = 0.38, 0.61; *P* < 0.0001) and verbal episodic memory composites (HR = 0.29; 95% CI = 0.23, 0.36; *P* < 0.0001) (see Table 3). Overall, higher cognitive function was associated with lower risk of MCI, probable dementia combined, and MCI or probable dementia, in particular the verbal and visual memory domains.

The measure of IAV at baseline, IAV Cognitive Domains, was a significant predictor of risk for probable dementia (HR = 4.72; 95% CI = 1.76, 12.70; *P* = 0.002) but not MCI (HR = 1.07; 95% CI = 0.44, 2.59; *P* = 0.889) or MCI or probable dementia (HR = 1.71; 95% CI = 0.82, 3.55; *P* = 0.152) (Table 3). IAV Cognitive Domains predicted incident dementia after controlling separately for overall cognition (HR = 4.15; 95% CI = 1.56, 11.0; *P* = 0.004) and EF (HR = 6.46; 95% CI = 2.37, 17.60; *P* = 0.0003) but not verbal episodic memory (HR = 2.19; CI = 0.71, 6.73; *P* = 0.17). Higher intraindividual variability among the cognitive domains at baseline, partially attributable to verbal episodic memory, increased the risk for probable dementia.

IAV Time significantly predicted risk of MCI (HR = 3.13; 95% CI = 2.05, 4.78; *P* = 0.0001) (Table 3). In the final model controlling for SD at baseline, EF, verbal episodic memory, overall cognition, EF slope, verbal episodic memory slope, and overall cognition slope, the results were slightly attenuated but still significant (HR = 2.32; 95% CI = 1.46, 3.69; *P* = 0.0004).

IAV Time also predicted the incidence of probable dementia (HR = 3.80; 95% CI = 2.23, 6.46; *P* = 0.0001), but in the final model results were fully attenuated (HR = 1.46; 95% CI = 0.84, 2.55; *P* = 0.18), largely by episodic verbal memory and episodic verbal memory slope. Lastly, IAV Time significantly predicted MCI or probable dementia (HR = 3.05; 95% CI = 2.13, 4.36; *P* = 0.0001). In the final model controlling for SD at baseline, EF, verbal episodic memory, overall cognition, EF slope, verbal episodic memory slope, and overall cognition slope, the results were slightly attenuated but still significant (HR = 1.70; 95% CI = 1.16, 2.49; *P* = 0.006). Overall, controlling for important cognitive variables, IAV Time predicts risk of MCI and MCI or probable dementia above and beyond baseline inter- and intraindividual differences in cognitive function.

## 4. Discussion

In a large cohort of older women, higher domain-specific cognitive function was associated with a decreased risk of MCI and a decreased risk of probable dementia. After adjustment for all other cognitive domains, deficits in verbal memory and figural memory remained independently associated with increased risk. Additionally, higher scores on composite measures of overall EF, and episodic verbal memory also predicted a lower risk of MCI and a lower risk of probable dementia. The measure of intraindividual variability (IAV Cognitive Domains) was a predictor of probable dementia (but not MCI or both groups combined) even after adjusting for interindividual differences in overall cognition (similar to [19]) and EF. However, adjusting for interindividual differences in episodic verbal memory attenuated the contribution of IAV Cognitive Domains to dementia risk. This research supports the finding that high variability within individuals among cognitive domains may be a significant predictor of dementia, in addition to the between-person differences in global cognitive ability and episodic memory that are a hallmark of the disease [6, 46].

The longitudinal change in intraindividual variability among cognitive domains (IAV Time) appears to be particularly important and robust in signaling the risk of cognitive impairment and dementia, above and beyond baseline interindividual differences in multiple measures of cognition and IAV cognitive Domains, especially in the early stages. Adjusting for multiple measures (baseline differences in overall cognition, EF, and verbal episodic memory, the standard deviation among the cognitive tests, and longitudinal interindividual changes in overall cognition slope, EF slope, and verbal episodic memory slope) in the model of IAV Time did not decrease the risk of MCI or MCI or probable dementia combined. In contrast (but similar to the baseline model), the contribution of IAV Time to probable dementia risk was attenuated after adjusting for longitudinal interindividual changes in verbal episodic memory slope. One possible explanation for this last finding is that on average (SD), women took 3.7 (2.0) years to progress to probable dementia from the time of their baseline cognitive tests, suggesting that most of the changes in IAV Time had already occurred (perhaps resulting in a slope underestimation for the women with probable dementia). While increasing intraindividual variability within tasks has been observed cross-sectionally in aging and may be an indicator of early cognitive impairment (e.g., [4, 22]), the current study's emphasis on longitudinal within-person variability among multiple cognitive domains in women with MCI and probable dementia is unique and shows that IAV time is also an indicator of risk for dementia, especially in the early stages.

One hypothesis that has been posited to explain normal age-related cognitive changes due to intraindividual variability in domains such as memory and executive function is dedifferentiation [47–49]. An increase in the strength of the correlations between various cognitive processes or abilities has been linked to greater within-person variability as well as higher coactivation of different cognitive processes [50, 51]. For example, Papenberg et al. [49] examined the relationship between intraindividual differences in trial-to-trial variability on a measure of forgetting and dedifferentiated memory functions and found that the correlation between episodic memory and spatial working memory was considerably higher in the high than the low variability group. Along these lines, we found that the correlations between most of the cognitive domains were somewhat higher in the probable dementia than in the nonprobable dementia group (data not shown), with the exception of vocabulary and attention with other domains. Although prior studies have not examined the association between intraindividual variability and de-differentiation in persons with and without dementia, this study provides some support for the hypothesis. In addition, Magnetic resonance imaging (MRI) studies show structural changes linked to increased performance variability that include frontal gray matter lesions and white matter changes on MRI scans (volumetric decline, demyelination, and hyperintensities) due to age-related changes in cerebral bold flow, vascular injury, or neurological conditions such as AD [4, 52]. In our probable dementia cohort, white matter hyperintensities as well as volumetric decreases likely explain poorer cognitive performance and increased performance variability (e.g., [53]).

This study was limited to the WHISCA cohort, which consists of volunteers who were eligible to participate in a clinical trial of hormone therapy, and is not representative of the general population. The classification of probable dementia included multiple etiologies, and while the pattern of cognitive deficits may vary by etiology, we first ruled out group differences in the baseline model and then included all adjudicated cases of probable dementia due to the small sample size. The WHISCA cognitive battery did not include a measure of cognitive processing speed, which may have provided additional important information about cognitive decline in dementia. While IAV Time may be an important marker of cognitive decline, it may not be as easy to evaluate in the clinic as more traditional cross-sectional types of measures, because it requires multiple cognitive assessments; although increasingly, memory disorders clinics provide this.

Within-person variability across multiple tests and measurement occasions explains additional risk for cognitive impairment and dementia beyond that accounted for by interindividual variation in cognitive function and may contribute to the early prediction of dementia. Greater IAV among cognitive domains represents a 3.6 to 4.4 times increased risk of probable dementia, which was only attenuated by adjusting for mean differences in verbal episodic memory. Greater IAV across time predicts a 2.0 to 3.7 times increased risk for MCI, a 1.5 to 6.4 times increased risk for probable dementia, and a 1.6 to 3.1 times increased risk for both groups combined. Adjusting for mean slope decreases in verbal episodic memory only attenuated the contribution of IAV Time to risk of probable dementia.

By extension, these results may have practical implications at the level of the individual that could be further refined with more research. For example, cognitive test batteries that measure multiple domains are commonly administered in memory disorders and other clinics where within-person across test comparisons over time may easily reveal imbalances in test scores that signal preclinical dementia. Greater knowledge of intraindividual differences in cognitive domains, both cross-sectional as well as longitudinal, could be used for early detection and intervention. In particular, longitudinal change in within-person across domain cognitive test scores (keeping in mind that shifts in within-person variability precede changes in mean performance) could signal the early stages of dementia.

## Figures and Tables

**Table 1:** Means (SDs) of the raw and standardized scores for the cognitive measures at baseline, standardized scores for executive function, verbal episodic memory, overall cognition, IAV Cognitive Domains, and IAV Time for WHISCA participants by group.

Means (SDs)								
Cognitive domain	All (*N* = 2066)	No impairment (*N* = 1965)	MCI	*P* value	Probable dementia	*P* value	MCI or probable dementia (*N* = 101)	*P* value
PMA vocabulary	36.93 (9.580)	37.24 (9.420)	31.19 (10.56)	<0.0001	31.00 (10.44)	<0.0001	30.86 (10.64)	<0.0001
Letter fluency	40.03 (12.35)	40.18 (12.27)	37.47 (13.57)	0.0639	37.82 (14.50)	0.2050	37.15 (13.51)	0.0162
Category fluency	29.15 (6.215)	29.37 (6.168)	24.07 (4.922)	<0.0001	25.44 (6.298)	<0.0001	24.91 (5.589)	<0.0001
*BVRT	6.915 (3.675)	6.755 (3.579)	9.838 (4.122)	<0.0001	10.36 (4.483)	<0.0001	10.01 (4.151)	<0.0001
CVLT (learning)	29.03 (6.174)	29.34 (6.009)	23.05 (5.527)	<0.0001	22.00 (6.931)	<0.0001	23.03 (6.308)	<0.0001
CVLT (short-delay free recall)	8.587 (3.018)	8.756 (2.925)	5.365 (2.672)	<0.0001	4.822 (3.284)	<0.0001	5.287 (2.923)	<0.0001
CVLT (long-delay free recall)	9.334 (2.975)	9.488 (2.893)	6.203 (2.669)	<0.0001	6.022 (3.583)	<0.0001	6.337 (2.984)	<0.0001
Digits forward	7.484 (2.059)	7.524 (2.066)	6.608 (1.719)	<0.0001	6.978 (1.738)	0.0789	6.713 (1.751)	<0.0001
Digits backward	6.723 (2.022)	6.765 (2.029)	6.041 (1.716)	0.0025	5.867 (1.590)	0.0005	5.891 (1.696)	<0.0001
Card rotations	56.38 (26.94)	57.13 (26.81)	43.78 (24.26)	<0.0001	36.89 (26.25)	<0.0001	41.66 (25.30)	<0.0001
Finger tapping (dominant)	38.57 (7.561)	38.67 (7.566)	36.64 (7.242)	0.0237	38.00 (6.801)	0.5583	36.71 (7.245)	0.0113
Finger tapping (nondominant)	36.46 (6.417)	36.58 (6.419)	34.11 (6.080)	0.0012	35.32 (5.915)	0.1931	34.14 (5.955)	0.0002

Standardized total score (*z*-scores): verbal fluency, CVLT, digits forward and backward, and fine motor speed								
Verbal fluency	−0.046 (0.952)	−0.019 (0.945)	−0.633 (0.873)	<0.0001	−0.490 (1.009)	0.0010	−0.570 (0.932)	<0.0001
CVLT	0.024 (0.932)	0.076 (0.902)	−1.01 (0.813)	<0.0001	−1.11 (1.104)	<0.0001	−0.988 (0.926)	<0.0001
Digits forward and backward	−0.017 (0.993)	0.006 (0.997)	−0.446 (0.805)	<0.0001	−0.396 (0.718)	0.0006	−0.460 (0.787)	<0.0001
Fine motor speed	−0.102 (1.085)	−0.084 (1.085)	−0.456 (1.037)	0.0038	−0.246 (0.995)	0.3216	−0.449 (1.028)	0.0010

Standardized scores (*z*-scores): executive function, verbal episodic memory, and overall cognition								
Executive function	−0.038 (0.978)	−0.008 (0.976)	−0.661 (0.816)	<0.0001	−0.544 (0.862)	0.0003	−0.633 (0.839)	<0.0001
Verbal episodic memory	0.024 (0.932)	0.076 (0.902)	−1.01 (0.813)	<0.0001	−1.11 (1.104)	<0.0001	−0.988 (0.926)	<0.0001
Overall cognition	−0.128 (0.962)	−0.077 (0.938)	−1.10 (0.887)	<0.0001	−1.12 (0.906)	<0.0001	−1.11 (0.902)	<0.0001

IAV Cognitive Domains (standard deviation across cognitive domains at baseline)								
SD	0.806 (0.260)	0.804 (0.259)	0.811 (0.272)	0.8182	0.917 (0.288)	0.0042	0.843 (0.269)	0.1475

IAV time (slope of IAV Cognitive Domains across measurement occasions)								
Slope SD	0.003 (0.519)	−0.009 (0.512)	0.221 (0.600)	0.0017	0.281 (0.594)	0.0002	0.237 (0.604)	0.0001

Note. PMA vocabulary: primary mental abilities vocabulary (total correct  −  1/3 number incorrect); letter fluency: letter fluency (number correct); category fluency: category fluency (number correct); BVRT: Benton Visual Retention Test (total figures with errors); CVLT (learning): California Verbal Learning Test (number correct 3 list A trials); CVLT (short-delay free-recall): California Verbal Learning Test (number correct list A short-delay free recall); CVLT (long-delay free-recall): California Verbal Learning Test (number correct list A long-delay free-recall); digits forward: digit span forward (number correct); digits backward: digit span backward (number correct); card rotations: card rotations (total correct  −  total incorrect); finger tapping (dominant): finger tapping test (taps for dominant hand); finger tapping (nondominant): finger tapping test (taps for nondominant hand); *higher scores reflect poorer performance.

**Table 2:** Hazard ratios for the cognitive domains (alone and controlling for all other cognitive domains) predicting MCI, probable dementia, and MCI or probable dementia.

Cognitive domains	MCI (*N* = 74)			Probable dementia (*N* = 45)			MCI or probable dementia (*N* = 101)		
	HR	95% CI	*P* value	HR	95% CI	*P* value	HR	95% CI	*P* value
Vocabulary alone	0.58	[0.46, 0.73]	<0.0001	0.46	[0.34, 0.62]	<0.0001	0.53	[0.44, 0.65]	<0.0001
Verbal fluency alone	0.49	[0.37, 0.65]	<0.0001	0.53	[0.37, 0.74]	0.0003	0.52	[0.41, 0.66]	<0.0001
Figural memory alone	0.49	[0.40, 0.61]	<0.0001	0.39	[0.30, 0.51]	<0.0001	0.46	[0.38, 0.55]	<0.0001
Verbal memory alone	0.27	[0.21, 0.35]	<0.0001	0.22	[0.16, 0.31]	<0.0001	0.29	[0.23, 0.36]	<0.0001
Attention/Working memory alone	0.63	[0.48, 0.81]	0.0005	0.61	[0.44, 0.85]	0.0039	0.60	[0.48, 0.75]	<0.0001
Spatial ability alone	0.60	[0.45, 0.79]	0.0003	0.36	[0.24, 0.53]	<0.0001	0.52	[0.40, 0.66]	<0.0001
Fine motor speed alone	0.79	[0.64, 0.98]	0.0306	0.91	[0.69, 1.20]	0.4962	0.79	[0.66, 0.95]	0.0113
Vocabulary | others	0.91	[0.69, 1.18]	0.4655	0.70	[0.49, 1.00]	0.0486	0.82	[0.65, 1.04]	0.0955
Verbal fluency | others	0.87	[0.65, 1.17]	0.3608	1.39	[0.95, 2.03]	0.0899	1.00	[0.78, 1.29]	0.9967
Figural memory | others	0.74	[0.58, 0.95]	0.0186	0.68	[0.50, 0.92]	0.0119	0.71	[0.57, 0.87]	0.0014
Verbal memory | others	0.32	[0.24, 0.42]	<0.0001	0.26	[0.17, 0.39]	<0.0001	0.34	[0.27, 0.44]	<0.0001
Attention/Working memory | others	0.95	[0.70, 1.28]	0.7260	0.99	[0.66, 1.47]	0.9555	0.94	[0.72, 1.22]	0.6186
Spatial ability | others	0.91	[0.67, 1.23]	0.5267	0.66	[0.43, 1.02]	0.0639	0.84	[0.64, 1.10]	0.2003
Fine motor speed | others	0.83	[0.66, 1.04]	0.1039	0.86	[0.63, 1.18]	0.3436	0.83	[0.68, 1.00]	0.0549

**Table 3:** Hazard ratios for IAV Cognitive Domains and IAV time (alone and controlling for baseline and slope overall cognition, EF, and verbal episodic memory) predicting MCI, probable dementia, and MCI or probable dementia.

Parameter	MCI (*N* = 74)			Probable dementia (*N* = 45)			MCI or probable dementia (*N* = 101)		
	HR	95% CI	*P* value	HR	95% CI	*P* value	HR	95% CI	*P* value
Overall cognition	0.32	[0.25, 0.41]	<0.0001	0.25	[0.18, 0.35]	<0.0001	0.30	[0.24, 0.37]	<0.0001
EF	0.48	[0.37, 0.63]	<0.0001	0.50	[0.35, 0.70]	<0.0001	0.48	[0.38, 0.61]	<0.0001
Verbal episodic memory	0.27	[0.21, 0.35]	<0.0001	0.22	[0.16, 0.31]	<0.0001	0.29	[0.23, 0.36]	<0.0001
Standard deviation (SD) (IAV Cognitive Domains)	1.07	[0.44, 2.59]	0.8891	4.72	[1.76, 12.7]	0.0021	1.71	[0.82, 3.55]	0.1525
SD | overall cognition	1.09	[0.44, 2.71]	0.8534	4.15	[1.56, 11.0]	0.0043	1.72	[0.82, 3.61]	0.1543
SD | EF	1.23	[0.50, 3.03]	0.6596	6.46	[2.37, 17.6]	0.0003	2.06	[0.98, 4.34]	0.0567
SD | verbal episodic memory	0.45	[0.18, 1.12]	0.0865	2.19	[0.71, 6.73]	0.1727	0.77	[0.36, 1.66]	0.5011
Slope standard deviation (SD) (IAV Time)	3.13	[2.05, 4.78]	<0.0001	3.80	[2.23, 6.46]	<0.0001	3.05	[2.13, 4.36]	<0.0001
Slope SD | overall cognition	2.99	[1.98, 4.51]	<0.0001	3.96	[2.37, 6.61]	<0.0001	3.10	[2.18, 4.41]	<0.0001
Slope SD | EF	3.03	[2.04, 4.52]	<0.0001	4.27	[2.45, 7.43]	<0.0001	3.07	[2.17, 4.34]	<0.0001
Slope SD | verbal episodic memory	3.27	[2.14, 5.00]	<0.0001	4.87	[2.83, 8.38]	<0.0001	3.35	[2.35, 4.78]	<0.0001
Slope SD | overall cognition, overall slope	2.34	[1.65, 3.31]	<0.0001	2.26	[1.51, 3.38]	<0.0001	2.07	[1.53, 2.80]	<0.0001
Slope SD | EF, EF slope	3.05	[2.04, 4.57]	<0.0001	3.76	[2.37, 5.96]	<0.0001	2.89	[2.11, 3.97]	<0.0001
Slope SD | verbal episodic memory, verbal episodic memory slope	2.05	[1.34, 3.15]	0.0010	1.50	[1.01, 2.23]	0.0433	1.58	[1.16, 2.16]	0.0039
Slope SD | SD, overall cognition, EF, verbal episodic memory, overall slope, EF slope, verbal episodic memory slope	2.32	[1.46, 3.69]	0.0004	1.46	[0.84, 2.55]	0.1842	1.70	[1.16, 2.49]	0.0061
